# Child and Family Characteristics Associated with Symptoms of Anxiety in Autistic Children: A Biobank Study

**DOI:** 10.1007/s10803-024-06706-7

**Published:** 2025-01-08

**Authors:** Willow J. Sainsbury, Andrew J. O. Whitehouse, Lisa Woods, Terence Jiang, Hannah Waddington

**Affiliations:** 1 Faculty of Education and Social Work, University of Auckland, NZ Office 117 A Building 114 3a Symonds St Auckland, New Zealand, 1010 New Zealand; 2 Telethon Kids Institute, Perth, Australia; 3 School of Mathematics and Statistics, Victoria University of Wellington, Wellington, New Zealand; 4 Affiliated with School of Psychology, Victoria University of Wellington, Wellington, New Zealand; 5 Faculty of Education, Victoria University of Wellington, Wellington, New Zealand

**Keywords:** Anxiety, Sleep, Autism, Parent Characteristics

## Abstract

Purpose: Autistic children have an increased likelihood of anxiety, but more research is needed on the characteristics that predict various types of anxiety in this population. Methods: In this study, we examined a range of child and family predictors of various types of anxiety using a sample of 452 autistic children from the Australian Autism Biobank. We used logistic regression to examine child and family predictors of four common types of anxiety in autistic children: generalised, phobic, separation, and social anxiety. Results: We found that 62.8% of children in this sample had symptoms of at least one type of anxiety. Poor quality sleep habits were the only predictive factor consistently identified across all anxiety symptom types. Specific to children with indicated generalised, separation, and phobic anxiety symptoms were the predictive factors of being older than five years, and specific to generalised and social anxiety were the predictive factors of higher cognitive abilities. Maternal anxiety was also a predictive factor in indicated children’s separation anxiety. Conclusion: These findings can help inform the provision of more targeted support for autistic people, particularly the interaction of poor sleep habits and anxiety symptoms.

Anxiety is formulated in multiple ways in the Diagnostic Statistics Manual (hereafter DSM-5) (American Psychiatric Association, 2013), with distinct diagnoses depending on the content and source of the anxious thought, the relationship to fear, and the person’s reaction (Pandolfi & Magyar, 2016). Types of anxiety included in the DSM-5 include generalised anxiety, separation anxiety, phobias, and social anxiety (American Psychiatric Association, 2013). There is research to suggest that autistic people may experience anxiety differently from non-autistic people (Bellini, 2006; Ben-Itzchak et al., 2020; Jenkinson et al., 2020). Regardless of type, the presence of anxiety has a significant negative impact on family life and academic outcomes (Adams & Emerson, 2021) and reduces participation in everyday and social activities for autistic children (Ambrose et al., 2022). Co-occurring anxiety symptoms are an essential area of study due to the increased prevalence and the significant impact on autistic people.

While not inherent to an autism diagnosis, autistic people have an estimated 42% greater chance of developing an anxiety disorder compared to their non-autistic peers (Hollocks et al., 2019). Across studies, autistic individuals have an elevated likelihood of anxiety compared to a neurotypical comparison group (Jolliffe et al., 2023) and a group of non-autistic children referred for clinical diagnoses (van Steensel & Heeman, 2017). The prevalence of different types of anxiety may vary depending on an individual’s age and whether they are autistic. It is estimated that one in five autistic children are diagnosed with anxiety disorder with the most common being a phobia, followed by social and generalised anxiety types (Thiele-Swift & Dorstyn, 2024; van Steensel et al., 2011). In contrast to this order of most common anxiety diagnoses, a study of adolescent autistic teenagers found separation anxiety to be the most prevalent (Ben-Itzchak et al., 2020). This pattern contrasts with a non-autistic population study where separation anxiety was associated with a younger age group (Ben-Itzchak et al., 2020). Social anxiety in an autistic population has been linked to differences in social communication and studies report a higher prevalence than a non-autistic population (Bellini, 2006; Jenkinson et al., 2020).

A variety of child and family characteristics may predict likelihood of anxiety in autistic and non-autistic children. Grondhuis and Aman (2012) examined the assessment of anxiety in autism research between 2000 and 2011. Of the 60 included articles, there were 36 different measures of anxiety used. Although there is no gold standard measure of anxiety in an autistic population, key factors include that the assessment is designed specifically for an autistic population and that it measures the particular types of anxiety (Grondhuis & Aman, 2012). A more recent meta-analysis by van Steensel and Heeman (2017) found 29 different anxiety measures used across 85 included articles, which described child characteristics that potentially increase the likelihood of anxiety in an autistic population. While results across these included studies were inconsistent, the authors concluded that higher cognitive abilities and increasing age were the most consistent predictors of the likelihood of anxiety (van Steensel et al., 2011). These characteristics are also predictive of anxiety in non-autistic children but appear to play a more significant role for autistic children compared with non-autistic children (van Steensel & Heeman, 2017). This increase in anxiety as children age is proposed to be due to increasing social demands and increasing awareness of symptoms of anxiety in an older population (van Steensel & Heeman, 2017). It has also been suggested that the association between higher cognitive abilities and anxiety might be due in part to the verbal articulation needed to describe the characteristics necessary for a diagnosis of a particular type of anxiety (Kerns et al., 2021). In a longitudinal study, these results were given specificity in relation to anxiety types with lower cognitive results predictive of higher separation anxiety and higher cognitive results predictive of higher generalised anxiety in autistic adolescence (Ben-Itzchak et al., 2020).

Familial and socio-demographic characteristics may also be predictive of anxiety in non-autistic children, however the current research remains focused on child factors in an autistic population (Behzadpoor et al., 2021; van Steensel & Heeman, 2017). Specifically, parental anxiety and depression and lower socioeconomic status are associated with higher anxiety in a non-autistic population (Moffitt et al., 2007). In addition, parental anxiety is also associated with higher anxiety in a non-autistic population, although depending on the anxiety type, the mechanism by which this occurs is unclear (Burt, 2009; Scaini et al., 2014). For example, heritability and non-shared environmental factors, such as peer group, were strongly predictive of social anxiety (Meier & Deckert, 2019; Scaini et al., 2014), while shared environmental factors and parental beliefs about anxiety were predictive of general anxiety symptoms (Manley & Francis, 2022). There is research on the high prevalence of anxiety in parents of autistic children, with 33% of parents reporting an anxiety disorder (Schnabel et al., 2019). Furthermore, in a study examining the negative interference of anxiety in the lives of autistic children, the variables that explained the most interference were the parents’ level of anxiety and difficulties with uncertainty (Adams & Emerson, 2021).

Given the main focus on child characteristics within the autism anxiety research (van Steensel & Heeman, 2017), there is considerable need for more research into possible family characteristics as predictors of anxiety in autistic children. There is also need for more research into the relative contribution on child and family factors to anxiety in autistic children. Therefore, this study builds on previous research by examining a wide range of child characteristics (gender, age, adaptive behaviour, autism characteristics, cognitive abilities, comorbid diagnoses, and sleep habits), parent (anxiety, depression, and autistic-like traits), and socioeconomic factors (parent education level) as predictors of different types anxiety in a large sample of children from the Australian Autism Biobank (Alvares et al., 2018). The specific types of anxiety examined in this study are generalised, social, separation, and phobic symptoms.

## Methods

### Participants and Setting

Participants were drawn from the Australian Autism Biobank (hereafter Biobank) (Alvares et al., 2018), which was conducted across four settings: Telethon Kids Institute (Perth); the Olga Tennison Autism Research Centre (Melbourne); the University of New South Wales (Sydney); and Lady Cilento Children’s Hospital (Brisbane) (Alvares et al., 2018). Ethical approval was provided by human ethics committees in all respective settings (Alvares et al., 2018). This approval was deemed sufficient by the Victoria University of Wellington Human Ethics Committee. Participants were children with a diagnosis of autism living in Australia aged between 2 and 17 years old and their families who completed relevant measures within the Biobank. Data collection began in 2013 and ceased in June 2018. (Alvares et al., 2018).

### Procedures

Access to data was granted by the Operations Committee of Australian Biobank, which comprises representatives of the autistic community, researchers, and the Co-operative Research Centre for Living with Autism (CRC) (Alvares et al., 2018). Caregivers (hereafter parents) gave informed consent for both themselves and their child to participate. Children over the age of 7 were given the option to provide written or verbal consent if they could understand the study requirements (Alvares et al., 2018). Researchers trained for reliable coding examined autistic children’s adaptive behaviour using the Vineland II (Sparrow et al., 2005), cognitive abilities using the Mullen Scale of Early Learning (MSEL) (Mullen, 1995) or the Wechsler Intelligence Scale for Children (WISC IV) (Wechsler, 2003) depending on age, sensory needs using a Short Sensory Profile- 2 (Dunn, 2014), and autism characteristics using The Autism Diagnostic Observation Schedule-2 (ADOS) (Lord et al., 2012). The Developmental, Dimensional and Diagnostic Interview (hereafter 3Di; (Skuse et al., 2004) was also administered by researchers as a semi-structured interview with the parents. Finally, parents filled out a bespoke Family Health Questionnaire (FHQ) (Alvares et al., 2018). The participants were eligible for inclusion from the Australian Autism Biobank if their parents completed the 3Di and FHQ.

### Measures

#### Family History Questionnaire

The Family History Questionnaire (FHQ) (Alvares et al., 2018) provided data for autistic children’s characteristics, including age, gender, and co-occurring diagnoses. Parents were able to select from a variety of co-occurring child diagnoses (e.g., global developmental delay, epilepsy, head trauma) and were able to select “other” to detail diagnoses that were not on this list. This variable was coded to dichotomously indicate the presence of at least one co-occurring diagnosis over the full range of diagnoses.

Family characteristics on the FHQ included maternal and paternal anxiety, depression, ethnicity, and highest level of education, as well as family income. Anxiety and depression were measured using dichotomous questions about whether the biological mother or father had ‘ever been told by a doctor that she/he had “anxiety disorder” or “major depression.“’.

Ethnicity was recorded as the predominant ethnic background of the participant with the option of Caucasian; Asian; Aboriginal or Torres Strait Islander; Māori or Pacific Islander, and Other. Total family income before tax was rated using 13 categories, incrementally increasing from $1AUD in $AUD8,000 increments to the highest category being “$104,001 and above.”

#### The Autism Diagnostic Observation Schedule-2

The most appropriate module for the child’s age and language ability was administered across five domains. A standardised comparison score across domains was subsequently derived using an algorithm relative to the module, age, and language ability of the autistic child (Lord et al., 2012). Higher scores indicate greater autistic characteristics (Lord et al., 2012). The internal consistency is given for each domain (communication, α = 0.74–0.84; social α = 0.86–0.91; communication/social interaction α = 0.91–0.94; stereotyped behaviours and restricted interests α = 0.63–0.65) (Lord et al., 2000).

#### Vineland Adaptive Behaviour Scale 2nd Edition

Parents completed four domains (Communication, Daily Living Skills, Socialisation, and Motor skills, if relevant) on the adaptive behaviour of their autistic child (Sparrow et al., 2005). The sum together gave an Adaptive Behaviour Composite Score relative to the child’s age (Sparrow et al., 2005). The internal consistency for the domains and adaptive behaviour composite score were between high 0.80s to low 0.90s (Sparrow et al., 2005).

#### Cognitive Abilities

Children aged 2–6 years of age were administered the Mullen Scale of Early Learning (MSEL), which measured four scales to produce a cognitive functioning estimate (M = 100, SD = 15) (Mullen, 1995). Mullen Scale of Early Learning (MSEL) internal consistency for each of the MSEL subscale ranges were between 0.95 and 0.97 (Mullen, 1995).

Children aged above six years were administered a Weschler Intelligence Scale for Children (WISC 4th edition), which measured four domains to produce a full-scale IQ estimate (M = 100, SD = 15) (Wechsler, 2003). The results for all ages were combined for one variable of cognitive abilities across ages (Alvares et al., 2018). The WISC 4th edition internal consistency score for full-scale IQ score reported a Cronbach’s alpha of 0.97 (Wechsler, 2003).

#### Children’s Sleep Habits Questionnaire

The Children’s Sleep Habits Questionnaire (CSHQ) was embedded in the FHQ (Owens et al., 2000). The Questionnaire had 34 questions about the nature of sleep and habits and was scored “rarely,” “sometimes,” “usually,” with a higher composite score representing poorer sleep and sleep habits. Internal consistency scores were reported at 0.78, with some criticism of the diagnostic ability of the tool with regards to sleep disorders (Markovich et al., 2014).

#### Autism Spectrum Quotient

Maternal and paternal autistic-like traits were collected using the autism spectrum quotient embedded in the FHQ (Baron-Cohen et al., 2001). This was calculated from 50 questions about the thinking style of the respective parent on a four-point Likert scale from “Definitely agree” to “definitely disagree” (Baron-Cohen et al., 2001). A composite score was then calculated, with the higher score representing a greater autism spectrum quotient (Baron-Cohen et al., 2001). Internal reliability for the composite score was reported at a Cronbach’s alpha of 0.97 (Baron-Cohen et al., 2001).

#### Short-Sensory Profile (2nd Addition)

The sensory score from a 34-item parent reported measure was used as a measure of responding to sensory input (Alvares et al., 2018). This was calculated across the four measured domains of sensory seeking, avoiding, sensitivity and registration (Alvares et al., 2018). Internal consistency of the total and subscale scores ranged from 0.70 to 0.90 (McIntosh et al., 1999).

#### The Developmental, Dimensional and Diagnostic Interview (3Di)

The 3Di is based on the Autism Diagnostic Interview-Revised ADI-R (Lord et al., 1994) with relevant sections on co-morbidities according to DSM − 5 symptom scales (Alvares et al., 2018). In the 3Di, there were six specific questions on generalised anxiety, four questions on phobic anxiety, eight questions on separation anxiety, and four on social anxiety. There were two questions on agoraphobic anxiety. All questions were scored on a three-point Likert-type scale (No (0), Possibly (1), and Definitely (2) and were combined across questions to calculate a dichotomous measure of the presence or absence of each type of anxiety. Each anxiety variable was coded dichotomously to indicate the presence or absence of at least one parent-reported anxiety symptom within that category. Participants received a score of “0” or “no anxiety symptoms” if they did not indicate that any anxiety was present across completed questions or ‘possibly’ was the only indication within that category. If a participant responded positively to a quarter or more of questions and had responded “definitely” that an anxiety symptom was present within that category, they received a score of “1”. On the 3Di, if the parent reported that the child experienced the anxiety-type symptoms, there was a selection question about how this symptom physically manifested for their autistic child, such as “difficulty breathing” or “nausea.” These questions were not included in the analysis due to parents having no option to indicate whether this section was not completed or non-applicable and the nature of conflating this section asking about a child’s response to anxiety with the previous questions indicating a presence of an anxiety symptom using a Likert scale. Interrater reliability for anxiety measures on 3Di and specific diagnostic categories was 96% agreement (‘κ for absolute agreement versus non-agreement was.0.92’) and diagnostic test-retest reliability agreement was 94% (‘κ for absolute agreement versus non-agreement was.0.89) (Skuse et al., 2004).

### Data Analysis and Management

Data were collated in Excel and exported to IBM SPSS version 29. The child variables included in the analysis were gender, age, presence of co-occurring diagnosis, CSHQ sleep score, ADOS-2 score, cognitive score, and Vineland composite adaptive behaviour scores. Parent variables included maternal and paternal anxiety and depression, and the socioeconomic status as indicated by the parent’s highest level of education. Parental education data was collapsed into five categories from seven categories, with the three categories combined into “less than 12 years of school” due to the small number of participants in the earlier school exit points (Waddington et al., 2020). The lack of delineation in the highest category for family income meant that this was not included as a predictor variable, and a better predictor of socioeconomic status was taken to be the variables of parental education (Davis-Kean et al., 2021). The 13 categories of family income were used as descriptive statistics only and divided into four categories, < $AUD 50,000, $50,001-100,004, and > $104,000.

Ethnicity and income are included as descriptive statistics but were not included as predictive variables. The dependent variables were the types of anxiety, which included generalised, phobic, separation, and social anxiety symptoms. The agoraphobic type of anxiety was removed from the analysis due to the lack of participation and the limited number of questions.

Due to the number of variables examined in this research and the corresponding time required for parents to complete these measures, there was substantial missing data across both child and family variables and the types of anxiety symptoms. To ensure a more complete data set, child and family variables that were not completed by 85% of participants were excluded from the analysis. This resulted in the exclusion of child sensory needs (complete for 81% of participants, data missing for 105 participants), paternal autistic traits (complete for 75% of participants, data missing for 194 participants) and maternal autistic traits (complete for 83% of participants, data missing for 94 participants). Each predictor variable was also categorically separated into four quartiles with an additional missing data category to enable direct comparisons across variables while allowing for missing data. The quartiles were decided according to the data distribution, with an approximately even number of participants across the quartiles.

To be included in analyses, participants must also have completed at least 75% of the questions on each anxiety symptom measure and at least 75% of the child and family variables. The included and excluded data sets were then statistically compared using a chi-squared test for each child and family variable and anxiety symptom outcome to check there no significant pattern of differences between individuals included and excluded from analysis, which might suggest a lack of external validity to a similar autistic population.

Chi-squared tests were used to examine the relationship between each child and family variable and anxiety type. The child and family variables which were significant at *p* ≤.01 in these chi-squared tests were then used as predictor variables in a binary logistic model for each anxiety type. If a predictor variable was significant to the level of 0.01 during the chi-squared tests for any one of the anxiety types, it was used for all anxiety types to create a consistent model.

## Results

### Sample Characterisation

Participant demographic information is presented in Table 1. The number of participants was reduced from 553 to 452 due to the exclusion of those who exceeded the 75% threshold for missing data. Chi-squared analyses indicated two significant differences between the included and excluded participant groups across all child, family and anxiety symptom variables. The included group was more likely to have autistic children with at least one reported co-occurring diagnosis (*X*^2^ (1) = 8.3, *p* =.004) and mothers with more indicated-chronic depression (*X*^2^ (1) = 7.4, *p* =.007).

**Table 1:** Summary of child, parent, and socioeconomic characteristics

Characteristic	*n* (%)/mean (SD)	Missing Data (*n*)
**Child Characteristics**		
Gender		
Male	362 (80.1%)	
Female	90 (19.9%)	
Age	6.9 years (SD = 3.84)	11
0–5	208 (46%)	
6–12	189 (41.8%)	
13–18	44 (9.7%)	
At least 1 co-occurring diagnosis	204 (45.1%)	27
CSHQ sleep score^+^	51.12 (SD = 10.36)	12
≤ 42.99 (best sleep)	103 (22.8%)	
43–49.99	122 (27%)	
50–58.99	109 (24.1%)	
≥ 59 (poorest sleep)	106 (23.1%)	
ADOS 2 Composite Score	7.11 (SD = 1.66)	19
≤ 5 (lowest autism characteristics)	55 (12.2%)	
6	94 (20.8%)	
7	101 (22.3%)	
≥ 8 (highest autism characteristics)	183 (40.5%)	
Cognitive Score	78.64 (SD = 23.52)	68
≤ 53 (lowest cognition)	91 (20.1%)	
54–80	101 (22.3%)	
81–97	94 (20.8%)	
≥ 98 (highest cognition)	98 (21.7%)	
Vineland Composite Score	73.93 (SD = 14.74)	46
≤ 63 (lowest adaptive behaviour)	91 (20.1%)	
64–71	106 (23.5%)	
72–81	100 (22.1%)	
≥ 83 (highest adaptive behaviour)	109 (24.1%)	
**Parent Characteristics**		
Maternal Ethnicity*		29
Caucasian	323 (71.5%)	
Asian	56 (12.4%)	
Māori	6 (1.3%)	
Aboriginal/Torres Strait Islander	4 (0.9%)	
Other	34 (7.5%)	
Paternal Ethnicity*		34
Caucasian	341 (75.4%)	
Aboriginal	1 (0.2%)	
Asian	48 (10.6%)	
Māori	3 (0.7%)	
Other	25 (5.5%)	
Maternal anxiety disorder	106 (23.5%)	1
Paternal- anxiety disorder	41 (9.1%)	4
Maternal- major depression	105 (23.2%)	1
Paternal- major depression	59 (13.1%)	4
**Socioeconomic Characteristics**		
Maternal education		23
< 12 years	36 (8.0%)	
12 years	46 (10.2%)	
Trade	75 (16.6%)	
University	229 (50.7%)	
Other	43 (9.5%)	
Paternal education		41
< 12 years	56 (12.4%)	
12 years	63 (13.9%)	
Trade	92 (20.4%)	
University	186 (41.2%)	
Other	14 (3.1%)	
Family Income $AUD*		36
< 50,000	60 (13.3%)	
50,0001-104,000	131 (29.0%)	
> 104,001	161 (35.6%)	
Prefer not to say	64 (14.2%)	
Anxiety		
No anxiety symptoms	168 (37.2%)	
At least one anxiety symptom	284 (62.8%)	
Anxiety types^+^		
Generalised	194 (42.9%).	
Phobic	161 (35.6%).	
Separation	152 (33.6%)	
Social	171 (37.8%).	

* Descriptive of the sample only and not included in analyses

^+^ Cronbach’s Alpha indicated internal consistency for CSHQ sleep measure (34 items, α = 0.88) and for each anxiety: Generalised (6 items, α = 0.96); Phobic (4 items, α = 0.95); Separation (8 items α = 0.77); Social (4 items, α = 0.87)

### Associations Between Individual Child and Family Variables and Anxiety Symptoms

See Table 2 for the results of the chi-square analyses for associations between child, parent, and socioeconomic variables and different types of anxiety symptoms.

**Table 2:** Chi-square comparison of child, parent, and socioeconomic predictors with types of anxiety

Characteristic	Generalised Anxiety Symptoms	Phobic Anxiety Symptoms	Separation Anxiety Symptoms	Social Anxiety Symptoms
X^2^(DF, *N* = 452) = Chi-square Value, p = *p-value*				
Child Characteristics				
Gender	(1) = 2.30 *p* =.129	(1) = 5.98 *p* =.014	(1) = 2.04 *p* =.153	(1) = 2.85 *p* =.091
Age	(3) = 37.37 ***p*** **<.001**	(3) = 13.43 ***p*** **=.004**	(3) = 39.76 ***p*** **<.001**	(3) = 10.83 *p* =.013
Co-diagnosis indicated	(2) = 3.50 *p* =.173	(2) = 2.22 *p* =.330	(2) = 0.77 *p* =.680	(2) = 0.25 *p* =.883
Sleep CSHQ score	(4) = 19.07 ***p*** **<.001**	(4) = 20.42 ***p*** **<.001**	(4) = 52.59 ***p*** **<.001**	(4) = 30.92 ***p*** **<.001**
ADOS 2 Composite Score	(4) = 8.45 *p* =.065	(4) = 5.75 *p* =.218	(4) = 4.70 *p* =.320	(4) = 0.26 *p* =.992
Cognitive Score	(4) = 30.50 ***p*** **<.001**	(4) = 6.48 *p* =.166	(4) = 24.43 ***p*** **<.001**	(4) = 20.11 ***p*** **<.001**
Vineland Composite Score	(4) = 3.47 *p* =.483	(4) = 12.61 *p* =.013	(4) = 0.38 *p* =.984	(4) = 1.15 *p* =.887
Parent Characteristics				
Mother-indicated anxiety disorder	(1) = 12.09 ***p*** **<.001**	(1) = 9.43 ***p =*** **.002**	(1) = 27.59 ***p*** **<.001**	(1) = 4.15 *p* =.042
Father-indicated anxiety disorder	(1) = 0.63 *p* =.472	(1) = 2.26 *p* =.133	(1) = 1.24 *p* =.265	(1) = 0.27 *p* =.869
Mother-indicated major depression	(1) = 4.10 *p* =.025	(1) = 4.99 *p* =.026	(1) = 3.29 *p* =.070	(1) = 0.965 *p* =.326
Father-indicated major depression	(1) = 4.69 *p* =.030	(1) = 6.86 ***p*** **=.009**	(1) = 2.33 *p* =.127	(1) = 0.60 *p* =.441
Socioeconomic Characteristics				
Maternal education	(5) = 6.68 *p* =.246	(5) = 6.27 *p* =.281	(5) = 4.13 *p* =.530	(5) = 3.65 *p* =.601
Paternal Education	(5) = 6.12 *p* =.295	(5) = 1.84 *p* =.871	(5) = 11.34 *p* =.045	(5) = 9.29 *p* =.098

Note. Significance set at *p ≤* 01

#### Child Characteristics

The chi-square comparison of child characteristics indicated that older children were also more likely to have generalised (*p* <.001), phobic (*p* =.004), separation (*p* <.001), and social anxiety symptoms (*p* =.013). Poorer sleep habits were also associated with an increased likelihood of all types of anxiety symptoms (*p* <.001). Higher cognitive scores were associated with an increased likelihood of generalised (*p* <.001), separation (*p* <.001), and social anxiety (*p* <.001) symptoms. There were no associations significant at the ≤ 0.01 level between any type of anxiety symptoms and indicated co-diagnoses, ADOS 2 composite scores, or Vineland composite scores.

#### Parental Characteristics

Maternal anxiety was associated with greater likelihood of generalized, separation (*p* <.001) and phobic anxiety symptoms (*p* =.002) in their autistic child. Paternal depression was associated with greater likelihood of phobic anxiety symptoms in their autistic child (*p* =.009). There was no significant association between paternal anxiety or maternal depression and child anxiety symptoms.

#### Socioeconomic Characteristics

There was no significant association between maternal and paternal highest level of education and symptoms of any type of child anxiety.

### Predictors of Symptoms of Child Anxiety

Separate binomial regression models were used to determine whether those child and family variables that were significant in the Chi-square analyses, that is age, sleep CSHQ scores, cognitive scores, and parent anxiety and depression were significant predictors of each type of anxiety symptom. Maternal and paternal measures are treated as pairs, thus, if one variable was significant, both measures were used in the model.

#### Generalised Anxiety Symptoms

The binomial regression model was statistically significant X^2^(15) = 77.286, *p* <.001. The model explained 21.1% (Nagelkerke R^2^) of the variance in parent-reported generalised anxiety and correctly classified 70.4% of cases. Children aged 6–12 had 2.47 times higher odds, and children aged 13–18 had 3.82 times higher odds than a 0–5 age group to have generalised anxiety symptom(s). Children with the worst sleep habits had 2.76 times higher odds than children with the best sleep habits to have generalised anxiety symptoms. Children with the lower category of IQ scores compared to average IQ scores (those in the range 81–97) had 3.43 times higher odds of having parent-reported generalised anxiety.

#### Phobic Anxiety Symptoms

The binomial regression model for phobic anxiety symptoms was statistically significant X^2^(15) = 46.030, *p* <.001. The model explained 13.3% (Nagelkerke R^2^) of the variance in parent-reported phobic anxiety and correctly classified 67.5% of cases. Children in the 6–12 age category had 2.05 times higher odds than children in the 0–5 age category of having parent-reported phobic-anxiety. Children with the worst sleep habits had 2.90 times higher odds than children with the best sleep habits to have parent-reported phobic anxiety.

#### Separation Anxiety Symptoms

The binomial regression model for separation anxiety symptoms was statistically significant X^2^(15) = 116.125, *p* <.001. The model explained 31.4% (Nagelkerke R^2^) of the variance in parent-reported separation anxiety and correctly classified 75.9% of cases. Children aged 6–12 and 13–18 had over 3 times higher odds than a 0–5 age group to have parent-reported separation anxiety. Children ranked with the worst sleep habits had 9.23 times higher odds than children with the best sleep habits to have parent-reported separation anxiety. Mothers, who reported a clinical diagnosis of anxiety were 2.78 times more likely to report separation anxiety symptoms in their children.

#### Social Anxiety Symptoms

The binomial regression model for social anxiety symptoms was statistically significant X^2^(15) = 54.851, *p* <.001. The model explained 15.6.% (Nagelkerke R^2^) of the variance in parent-reported social anxiety and correctly classified 64.4% of cases. Children ranked with the worst sleep habits had 4.71 times higher odds than children with the best sleep habits to have parent-reported social anxiety. Children with higher recorded cognition or missing data than the lowest cognitive data group, were more likely to have parent-reported social anxiety.

**Table 3:** Binary logistic regression of anxiety across significant (*p* ≤.01) predictors

	Generalised Anxiety		Phobic Anxiety		Separation Anxiety		Social Anxiety	
Predictors	*p*	OR	*p*	OR	*p*	OR	*p*	OR
Age								
6–12 years v. 0–5 years	**< 0.001**	2.474	**0.003**	2.049	**< 0.001**	3.343	0.174	1.383
13–18 years v. 0–5 years	**< 0.001**	3.822	0.167	1.679	**0.001**	3.569	0.28	2.252
Missing data v. 0–5 years	0.075	3.389	0.137	2.707	0.037	4.947	0.767	1.221
Sleep CSHQ Scores								
42–49.99 v. < 41 (best sleep)	0.072	1.735	0.297	1.394	0.018	2.422	0.037	1.943
50–59.99 v. < 41 (best sleep)	0.012	2.196	**0.008**	2.306	**< 0.001**	3.481	**0.006**	2.439
≥ 60 (poorest sleep) v. < 41 (best sleep)	**0.001**	2.764	**< 0.001**	2.906	**< 0.001**	9.231	**< 0.001**	4.710
Missing data v. < 41 (best sleep)	0.944	0.948	0.662	1.370	0.026	5.090	0.078	3.220
Cognitive group								
54–80 v. < 53 (lowest cog.)	0.049	2.005	0.833	0.932	0.135	1.842	0.025	2.193
81–97 v. < 53 (lowest cog.)	**< 0.001**	3.343	0.860	0.941	0.046	2.261	**0.004**	2.798
≥ 98 (highest cog.) v. < 53 (lowest cog.)	0.047	2.075	0.267	0.671	0.047	2.277	0.090	1.880
Missing data v. < 53 (lowest cog.)	0.030	2.390	0.690	1.165	0.077	2.216	**0.005**	3.048
Parent Anxiety								
Maternal anxiety disorder v. no anxiety	0.036	1.727	0.076	1.576	**< 0.001**	2.783	0.309	1.301
Paternal anxiety disorder v. no anxiety	0.499	0.753	0.907	0.953	0.876	0.932	0.646	1.210
Parent Depression								
Maternal depression v. no depression	0.919	1.027	0.544	1.168	0.338	0.761	0.597	0.871
Paternal depression v. no depression	0.222	1.539	0.039	2.034	0.532	1.270	0.935	1.029

Note. OR = Odds Ratio, cog. = cognition

## Discussion

This study examined whether a range of child and family characteristics were predictive of the presence of four types of anxiety symptoms in autistic children. The initial analysis indicated that child age, sleep habits, cognitive scores, and maternal anxiety and paternal depression were significantly and individually related to the presence or absence of an anxiety symptom type. These characteristics were then used in logistic regression analyses to determine their combined predictive effects for each anxiety symptom type. Better sleep habits were significantly associated with a lower chance of all anxiety symptom types, and younger children were less likely to have generalised, phobic and separation anxiety. Finally, children with higher-measured cognitive scores were more likely to have generalised anxiety and social anxiety compared to those with lower cognitive scores. Maternal reported diagnosis of an anxiety disorder was also associated with separation anxiety.

Poor quality sleep habits as described by The Children’s Sleep Habits Questionnaire (CSHQ) (Owens et al., 2000) was the only consistent significant predictor across all anxiety types. A bidirectional relationship between sleep and anxiety posits that sleep quality is reduced by anxiety, and anxiety symptoms increase with lack of sleep (Nguyen et al., 2022). Many autistic children also experience clinically significant sleep disturbance, with the suggestion that sleep difficulties might be related to some autism characteristics, such as transitional challenges, which might make quality sleep particularly challenging in this population (Vasa et al., 2020; Waddington et al., 2020). Findings in a non-autistic population also showed that pre-existing anxiety exacerbates the emotional effects of poor sleep, which might extend the implications of the relationship between anxiety, sleep and autism (Palmer & Alfano, 2020). The results of this study add to the literature in confirming poor quality sleeps association with anxiety and suggest that sleep is a critical variable for autistic children with anxiety.

The association between poor sleep, higher IQ, and older children showing specific anxiety is consistent with a meta-analysis of anxiety in an autistic populations (Vasa et al., 2020). The latter examples of younger and lower IQ children presenting with less anxiety might be a case of younger children and children with intellectual difficulties being less able to articulate anxiety symptoms (van Steensel & Heeman, 2017). There is also evidence that anxiety worsens with age and, therefore, becomes more apparent (Vasa et al., 2020). Concerns around older and more cognitively able autistic children being able to more easily articulate the nuanced symptomology of the anxiety types compared to younger and less cognitively able autistic children, particularly on parent-reported measures, is an important caveat in interpreting parent-reported data (Kerns et al., 2021; van Steensel & Heeman, 2017).

The prevalence of anxiety symptoms across this population of autistic youth ranged from 33.6% (separation anxiety) to 42.9% (generalised anxiety) with approximately two fifths of the participants definitely experiencing at least one symptom of anxiety. Estimating prevalence rates of anxiety in an autistic population is challenging with broad parameters given between 11 and 84% (Thiele-Swift & Dorstyn, 2024). This broad estimate is explained by the different natures of the study populations and clinically elevated anxiety symptoms versus diagnoses of anxiety (Thiele-Swift & Dorstyn, 2024). However, it is accepted that autistic children appear to have higher co-occurring anxiety compared to a non-autistic clinically-referred population (Thiele-Swift & Dorstyn, 2024; van Steensel et al., 2011). Bellini (2006) proposed that higher scores of social differences led to developing higher anxiety. More recently, Conner et al. (2020) suggested the mechanism by which this might occur in that social differences might amplify difficulties in emotional regulation or being able to modulate responses/behaviour, thus increasing anxiety in autistic populations. However, the relationship with increased autistic characteristics and anxiety was not noted in this sample as higher ADOS-2 scores were not predictive of anxiety in the initial statistical analysis.

Meta-analysises have found that parental anxiety is positiviely associated with anxiety in autistic children (van Steensel & Heeman, 2017; Vasa et al., 2020), which concurred with the findings of this study, however results were specific to the relationship between maternal anxiety and reported separation anxiety symptoms. One possible hypothsis for this relationship might be that there is an over-reporting of separation anxiety symptoms through the mediating effect of the mother’s own anxiety symptoms, however there is evidence to suggest that separation anxiety symptoms are often slightly under-reported by mothers of autistic children in comparison with other anxiety symptoms (Blakeley-Smith et al., 2012). Finally, there was no strong predictive association of the socio economic status (SES) measure of parental education level and anxiety symptoms. This result is aligned with a large study of a non-autistic population showing that SES is more strongly related to externalising diagnoses than depression or anxiety (Peverill et al., 2021). Regardless, in a USA population, Peverill et al. (2021) found that the “receipt of public assistance” followed by “parental education level” were the two measures of socio-economic status that most correlated with “psychopathology.” (Peverill et al., 2021).

This study is limited in several ways. The data set had significant missing data, and although the data set of the excluded and included participants were compared and a few differences identified, this remains a limitation. This study also examined a dichotomous measure of presence or absence of anxiety symptoms, which is a limitation compared to higher thresholds of clinically diagnosed anxiety types (Thiele-Swift & Dorstyn, 2024). Finally, a limitation of the current study is the reliance on parent-reported anxiety symptoms, as there has been some indication that autistic children and other informants might report these differently, with children able to indicate hidden anxiety levels on self-report measures (van Steensel & Heeman, 2017). Parent-reported accounts of anxiety tend to underestimate reports of internalising anxiety and might be biased towards children who can articulate or overtly signal their anxiety (Thiele-Swift & Dorstyn, 2024). This also makes measures of responses to anxiety difficult to quantify across participants such as the selection question on the 3Di about how anxiety symptoms physically manifest with a bias towards children who might show overt behaviour (Thiele-Swift & Dorstyn, 2024).

The models for phobic and social anxiety type also had relatively low Nagelkerke R^2^ values so it is possible that other factors beyond those measured in these studies would enhance these models. Future research might further investigate particular autistic characteristics, such as tolerance for uncertainty, as there is evidence that this might explain variance in autistic children’s anxiety presentation (Adams & Emerson, 2021). Anxiety has complex and multiple ways of presenting, and numerous factors likely to play a role in its presentation for autistic children.

In conclusion, the multifactorial nature of the co-occurrence of anxiety in an autistic population and the multiple types of anxiety present a challenge for researchers to robustly pinpoint possible characteristics that might influence anxiety presentation in autism. This study used a large Australian Autism Biobank participant group to examine possible influences. However, the robust nature of the relationship between poor-quality sleep habits and greater likelihood of anxiety across anxiety types in this sample suggests that this is an important finding. The implications of this finding include targeting sleep habits for autistic children with co-occurring anxiety. Further research to examine the interplay of anxiety, sleep and autism, including other co-occurring symptoms and interference effects, would help elucidate the impact of this relationship between anxiety and sleep in an autistic population.
